# Dose-response association between 24-hour total movement activity and testosterone deficiency in adult males

**DOI:** 10.3389/fendo.2023.1280841

**Published:** 2024-01-12

**Authors:** Shenghao Wu, Wu Chen, Yaoyao Cai, Weiting Xia

**Affiliations:** ^1^ Reproductive Medicine Center, Department of Obstetrics and Gynecology, The Second Affiliated Hospital and Yuying Children’s Hospital of Wenzhou Medical University, Wenzhou, Zhejiang, China; ^2^ Urology Department of Wenzhou People’s Hospital, The Third Affiliated Hospital of Shanghai University, Wenzhou, Zhejiang, China; ^3^ Department of Obstetrics, The First Affiliated Hospital of Wenzhou Medical University, Wenzhou, Zhejiang, China; ^4^ Department of Gynecology, The First Affiliated Hospital of Wenzhou Medical University, Wenzhou, Zhejiang, China

**Keywords:** male health, physical activity, testosterone deficiency, NHANES, accelerometer data

## Abstract

**Background and objectives:**

Previous studies on the relationship between physical activity and testosterone are limited and controversial. Hence we investigated whether high level of physical activity is associated with a low risk of testosterone deficiency (TD).

**Methods:**

This cross-sectional analysis was conducted in a representative sample of US adult males who participated in the 2011-2014 cycle of the National Health and Nutrition Examination Survey (NHANES). We used the monitor independent movement summary (MIMS) to assess activity intensity, a novel physical activity metrics developed using raw data collected by accelerometers. Multivariable regression and smooth curve fitting was used to describe the relationships between physical activity and TD, and segmented regression model were used to analyze the threshold effect between them. Sensitivity analysis was conducted using interaction and stratified analysis.

**Results:**

A U-shaped relationship between daily MIMS units and risk of TD was observed. The optimal value of daily MIMS units for the lowest risk of TD was 14.77 (×10^3^), the risk of TD decreased by 5% in patients per unit increase of daily MIMS units when daily MIMS units <14.77 (×10^3^) (adjusted OR = 0.95, 95%CI: 0.91, 0.99), but increased by 12% per unit increase of daily MIMS units when daily MIMS units ≥14.77 (×10^3^) (adjusted OR = 1.12, 95%CI: 1.01, 1.23). In sensitivity analyses, the threshold effect was also similar according to baseline characteristics (P-interaction >0.05).

**Conclusion:**

In a nationally representative sample of US adult males, light to moderate intensity physical activity is associated with a lower odds of TD, while high-intensity physical activity is associated with a higher risk of TD.

## Introduction

Keeping physically active is beneficial to men of all ages. It has been shown that physical activity can reduce mortality of cardiovascular disease and the risk of type 2 diabetes, and may even improve cognitive performance (1–3). However, the majority of US adults have not met the recommended physical activity level, which is at least 2.5 hours of more than moderate intensity physical activity each week (4).

Testosterone deficiency (TD), defined as a syndrome of low testosterone combined with symptoms such as decreased libido, sexual dysfunction and decreased energy (5), is a common disease that affects approximately one-third of middle-aged and older men, and the risk of incidence is strongly associated with advancing age (6). The deficiency of serum testosterone levels in males can lead to negative emotional states, fatigue, decreased libido and erectile dysfunction (ED). Additionally, TD is associated with changes in body metabolism, such as decreased bone mineral density, osteoporosis, decreased lean body mass, and increased fat mass (7–9). Therefore, TD has become a growing concern in the field of male reproductive health.

Previous studies have explored the association between physical activity and testosterone (10–13). Some of these studies used physical activity data derived from self-reported interview (10, 11), while others used accelerometer measurements to obtain the intensity of physical activity (12, 13). Due to the fact that accelerometer-derived metrics are usually device-specific, it is challenging to compare the results of studies using different accelerometers. Thus, researchers have used the raw data collected by the accelerometer to develop new metrics of physical activity, like the Monitor Independent Movement Summary (MIMS) unit, which are not specific (14). The objective of this study was to investigate the relationship between physical activity measured in MIMS units and testosterone deficiency in a nationally representative sample of US adults. We hypothesized that the level of physical activity among participants would not be associated with the risk of testosterone deficiency.

## Materials and methods

### Study participants

**Figure 1:**
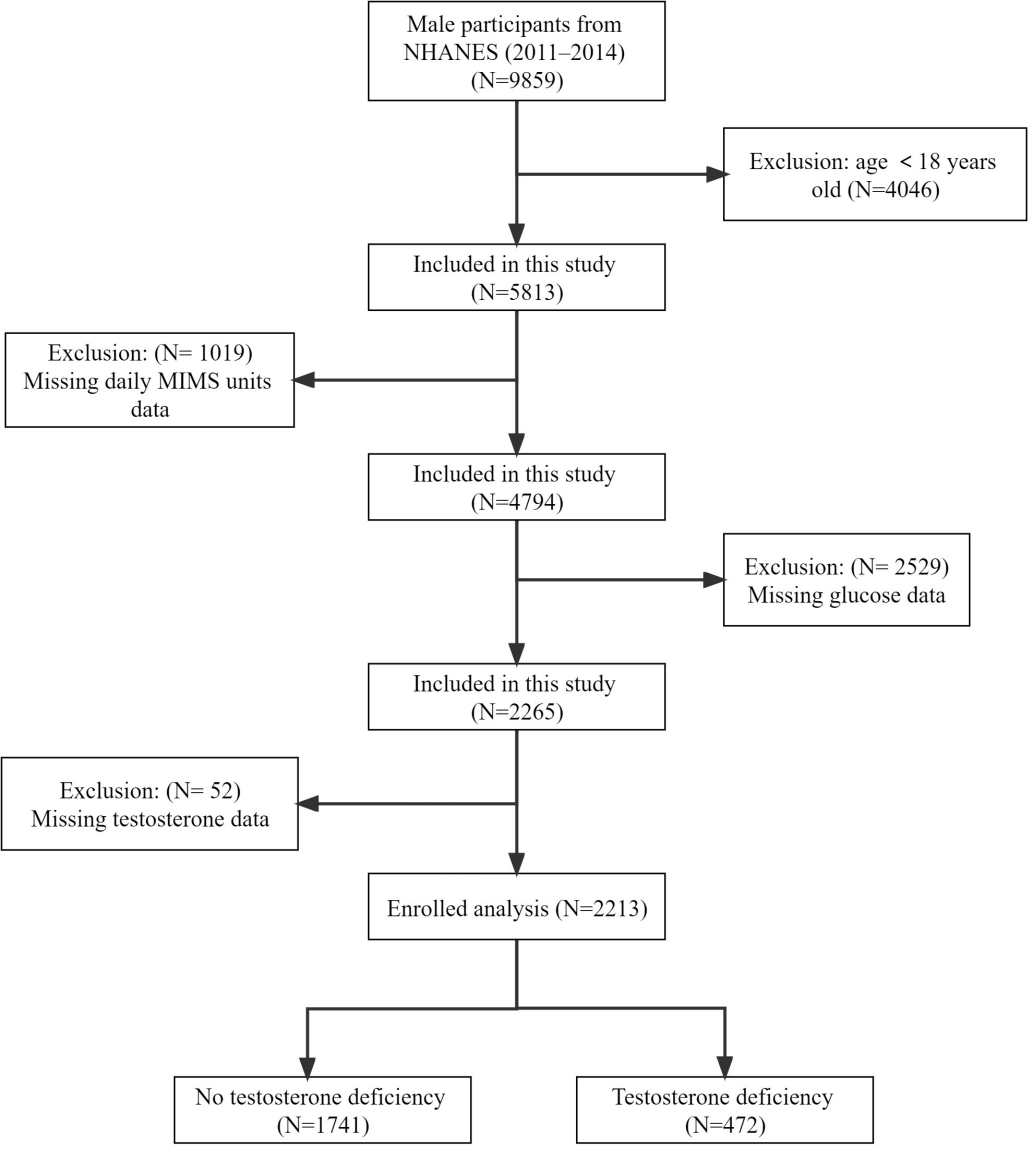
Flow chart of eligible participants’ selection.

The National Health and Nutrition Examination Survey (NHANES) was a series of national population-based surveys conducted by the National Center for Health Statistics (NCHS) to assess the health status of US citizens. Due to the complex multi-stage probability sampling design (counties, segments, households and individuals) utilized in the NHANES study, the participants included showed highly representative of the national population of US. Our study sample included 9859 male participants from the NHANES 2011-2014 cycles, who represented 150843141 males in the population. These cycles were selected because wrist accelerometry data are currently available only in 2011-2012 and 2013-2014. We excluded 4046 participants younger than 18 years, 1019 participants with missing physical activity data, 2529 participants with missing fasting glucose data, and 52 participants with missing testosterone data, finally we included 2213 participants for the final analysis (**Figure 1**).

### Informed consent

The Research Ethics Review Board of the NCHS approved the study of NHANES, and all study participants provided informed written consent. NHANES data were publicly accessible on the website (https://www.cdc.gov/nchs/nhanes/).

### Definition of physical activity (MIMS units) and testosterone deficiency

Activity was measured by the accelerometer (ActiGraph GT3X+, Pensacola, FL). Seven consecutive 24-hour periods were required for participants to wear the device on their non-dominant wrist. The device was programmed to obtain three-axis acceleration measurements at a sampling frequency of 80 Hz and was designed to be waterproof (15). The raw data from the accelerometer devices was collected and aggregated into MIMS units with 1-minute time frame through non-proprietary open-source and device-independent algorithms (14). Specifically, digital signal processing techniques are used to harmonize raw data from devices with different dynamic ranges and sampling rates to be able to capture normal human motion independently of the monitor, and then aggregating the raw data to generate a motion aggregation unit (MIMS unit). Method steps include raw signal coordination to eliminate differences between devices (such as dynamic g range, sampling rate), band-pass filtering (0.2-5.0 Hz) to eliminate non-human motion, and signal aggregation to reduce the data, ultimately allowing activity data to be simplified, visualized, and summarized (14). The total amount of movement activity for the 24-hour period was represented by the accumulation of the 1440 minute level MIMS units per day. In our analyses, the three-axis MIMS units were summed for all valid days and the average value was calculated.

Based on prior studies using wristband accelerometers to obtain data and the NHANES accelerometer protocol (16, 17), we excluded invalid days with less than 1440 minutes of wear time or with ≥ 17 hours of sleep in order to reduce measurement error. We took into account that previous studies have confirmed that one day is adequate for group-level analysis (18), so participants with a minimum of one valid wear day were included in our analysis.

In addition, according to the American Urological Association guidelines (19), testosterone deficiency was defined as less than 300 ng/dL of total testosterone.

### Definition of other variables

Blood collection was performed in the morning after fasting to collect total cholesterol, high-density lipoprotein (HDL), triglycerides, and fasting glucose. In-person home interviews were conducted to obtain demographic information, including age, race, marital status, education level, household income, smoking status, and drinking status. In our analysis, the poverty-to-income ratio (PIR) was used as a surrogate of household income and was divided into four groups: “0-1.3 RIP”, “1.3-3.5 RIP”, “> 3.5 RIP” and “Missing data”. Smoking status was divided into four groups based on the smoking frequency: “Every day”, “Some days”, “Not at all” and “Missing data”. Drinking status was divided into four groups based on daily alcohol consumption: “None or light drinker” (≤1 drinks per week), “Moderate drinker” (2-8 drinks per week), “heavy drinker” (>8 drinks per week), and “Missing data”. Hypertension and diabetes were obtained from the self-report of health questionnaire.

### Statistical analysis

In order to avoid oversampling minorities and thus provide a final unbiased and accurate estimate of the population effect, we adopted the adjusted analysis approach in which we used population-weighted parameters when investigating baseline characteristics and the association between physical activity level and risk of TD.

According to the quartiles of daily MIMS units (×10^3^), participants were divided into four groups: Q1 (<9.29), Q2 (9.29≤ ~ <11.88), Q3 (11.88≤ ~ <14.78), and Q4 (≥14.78). All continuous variables were presented as means (95% CI), and categorical variables were presented as percentage (95% CI). The difference between the four groups was examined by the Chi-square test or Kruskal-Wallis H test. We used logistic regression model to assess the association between physical activity and TD. In the analysis we developed three models, Model I was not adjusted, Model II was adjusted for age and race, and Model III was adjusted for age, race, BMI, HDL, glucose, hypertension, diabetes. We selected these confounders because they changed the estimates of daily MIMS units on TD by more than 10%. In the models, we also performed tests for linear by entering the median of each quartiles of daily MIMS units as a continuous variable.

In addition, we first used smooth curve fitting to examine whether the independent variable is partitioned into intervals with an adjustment for age, race, BMI, HDL, glucose, hypertension and diabetes. We applied segmented regression (piece-wise regression) that is using a separate line segment to fit each interval. Log-likelihood ratio test comparing one-line (non-segmented) model to segmented regression model was conducted to determine if threshold exists. The inflection point that connecting the segments was based on the maximum likelihood given by the model, which was determined using a two-step recursive method.

In sensitivity analyses, interaction and stratified analysis were conducted according to age, BMI status and Diabetes. EmpowerStats software (X&Y solutions, Inc., Boston MA) and R language 4.2.1 were used for statistical analysis in this study. The statistical difference was defined as two-tailed p-value below 0.05.

## Results

### Demographic and clinical characteristics of study participants

A total of 2213 individuals were enrolled in the study. The baseline characteristics of each group were shown in **Table 1**. We found that Q1 participants had the higher age 49.65 (47.79,51.51) years and highest prevalence of TD [27.10% (23.41%,31.14%)]. At the same time, they were more likely to be obese [39.59% (34.55%,44.86%)], and with complications such like hypertension [41.70% (36.50%,47.09%)] and diabetes [16.94% (13.59%,20.90%)]. Most variables were significantly different across the four groups (p <0.05). In addition, given the high number of missing values in the participant’s smoking data (approximately 50%), we removed this data from the baseline characteristics.

**Table 1:** Weighted demographic and clinical characteristics according to the quartiles of daily MIMS units.

	Q1	Q2	Q3	Q4	P-value
**Age (years)**	(515, 23776958) 49.65 (47.79,51.51)	(521, 24394231) 50.10 (48.43,51.77)	(527, 26145900) 43.99 (42.14,45.84)	(523, 25377294) 40.36 (38.98,41.73)	<0.0001
Race					<0.0001
Mexican American	(36, 23776958) 4.71 (2.93,7.51)	(52, 24394231) 6.48 (4.24,9.78)	(56, 26145900) 7.00 (4.18,11.51)	(116, 25377294) 16.96 (12.52,22.56)	
Other Hispanic	(34, 23776958) 4.36 (2.47,7.59)	(44, 24394231) 4.20 (2.73,6.41)	(60, 26145900) 7.02 (4.22,11.47)	(65, 25377294) 8.74 (5.88,12.80)	
Non-Hispanic White	(256, 23776958) 73.98 (66.82,80.06)	(235, 24394231) 70.89 (63.80,77.09)	(217, 26145900) 67.56 (61.60,73.01)	(186, 25377294) 58.78 (50.73,66.38)	
Non-Hispanic Black	(104, 23776958) 9.19 (5.90,14.04)	(96, 24394231) 9.75 (6.45,14.48)	(114, 26145900) 11.86 (8.88,15.67)	(101, 25377294) 11.34 (8.51,14.95)	
Other Race	(85, 23776958) 7.76 (5.53,10.79)	(94, 24394231) 8.68 (6.45,11.59)	(80, 26145900) 6.55 (4.60,9.25)	(55, 25377294) 4.19 (2.81,6.20)	
Marital status					0.1198
Married	(277, 23776958) 54.03 (47.75,60.18)	(314, 24394231) 63.33 (57.60,68.71)	(276, 26145900) 56.73 (51.54,61.78)	(263, 25377294) 53.91 (47.62,60.08)	
Other	(207, 23776958) 41.46 (35.84,47.31)	(187, 24394231) 33.53 (28.13,39.39)	(228, 26145900) 39.30 (34.24,44.61)	(230, 25377294) 42.19 (36.48,48.11)	
Missing data	(31, 23776958) 4.51 (3.08,6.56)	(20, 24394231) 3.14 (1.93,5.06)	(23, 26145900) 3.96 (2.15,7.18)	(30, 25377294) 3.90 (2.58,5.87)	
Education level					<0.0001
Less than high school	(103, 23776958) 16.81 (11.68,23.58)	(92, 24394231) 12.80 (9.71,16.68)	(100, 26145900) 11.12 (8.04,15.18)	(163, 25377294) 26.09 (22.06,30.57)	
High school or above	(381, 23776958) 78.68 (71.62,84.37)	(409, 24394231) 84.06 (80.34,87.19)	(404, 26145900) 84.92 (80.58,88.42)	(330, 25377294) 70.01 (65.59,74.08)	
Missing data	(31, 23776958) 4.51 (3.08,6.56)	(20, 24394231) 3.14 (1.93,5.06)	(23, 26145900) 3.96 (2.15,7.18)	(30, 25377294) 3.90 (2.58,5.87)	
Household income					<0.0001
0–1.3RIP	(164, 23776958) 24.71 (19.06,31.38)	(134, 24394231) 17.19 (12.22,23.65)	(163, 26145900) 21.08 (16.25,26.88)	(194, 25377294) 26.83 (22.74,31.35)	
> 1.3–3.5 RIP	(171, 23776958) 36.43 (31.04,42.18)	(157, 24394231) 29.07 (23.85,34.91)	(153, 26145900) 28.51 (23.01,34.73)	(186, 25377294) 37.39 (32.96,42.04)	
> 3.5 RIP	(147, 23776958) 32.80 (26.53,39.75)	(193, 24394231) 49.31 (40.81,57.84)	(170, 26145900) 44.78 (37.73,52.06)	(103, 25377294) 30.32 (25.43,35.70)	
Missing data	(33, 23776958) 6.07 (3.37,10.67)	(37, 24394231) 4.43 (2.78,7.00)	(41, 26145900) 5.63 (3.84,8.20)	(40, 25377294) 5.47 (3.57,8.28)	
BMI status					0.0088
Normal or low weight	(156, 23776958) 28.83 (24.71,33.32)	(149, 24394231) 25.34 (19.62,32.06)	(147, 26145900) 26.57 (21.41,32.47)	(165, 25377294) 26.90 (22.03,32.38)	
Overweight	(159, 23776958) 30.22 (25.32,35.63)	(200, 24394231) 39.92 (35.10,44.95)	(214, 26145900) 40.29 (34.07,46.85)	(217, 25377294) 44.41 (38.42,50.56)	
Obesity	(192, 23776958) 39.59 (34.55,44.86)	(168, 24394231) 34.14 (28.37,40.43)	(166, 26145900) 33.13 (26.95,39.96)	(141, 25377294) 28.70 (23.36,34.70)	
Missing data	(8, 23776958) 1.36 (0.47,3.84)	(4, 24394231) 0.60 (0.19,1.89)	(0, 26145900) 0.00 (0.00,0.00)	(0, 25377294) 0.00 (0.00,0.00)	
Drinking status					<0.0001
None or light drinker	(107, 23776958) 16.45 (12.75,20.96)	(124, 24394231) 27.27 (21.97,33.31)	(97, 26145900) 17.02 (12.95,22.05)	(74, 25377294) 14.88 (10.98,19.87)	
Moderate drinker	(201, 23776958) 46.64 (41.67,51.68)	(225, 24394231) 45.91 (41.08,50.81)	(269, 26145900) 55.78 (50.76,60.68)	(290, 25377294) 61.02 (55.70,66.08)	
Heavy drinker	(19, 23776958) 4.59 (2.56,8.10)	(15, 24394231) 2.96 (1.50,5.76)	(20, 26145900) 4.39 (2.31,8.19)	(24, 25377294) 3.91 (2.50,6.05)	
Missing data	(188, 23776958) 32.32 (26.98,38.16)	(157, 24394231) 23.86 (18.49,30.21)	(141, 26145900) 22.81 (17.41,29.30)	(135, 25377294) 20.19 (16.01,25.14)	
**Total cholesterol (mg/dL)**	(515, 23776958) 182.20 (177.90,186.50)	(521, 24394231) 187.29 (181.29,193.29)	(527, 26145900) 188.62 (183.00,194.23)	(523, 25377294) 187.21 (184.30,190.12)	0.1045
**HDL cholesterol (mg/dL)**	(515, 23776958) 46.07 (44.86,47.27)	(521, 24394231) 47.49 (45.70,49.28)	(527, 26145900) 48.51 (47.25,49.78)	(523, 25377294) 50.36 (48.73,51.99)	0.0005
**Triglyceride (mg/dL)**	(515, 23776958) 143.66 (132.06,155.25)	(521, 24394231) 138.35 (124.71,151.99)	(527, 26145900) 132.42 (122.71,142.12)	(522, 25320861) 126.06 (114.06,138.07)	0.0566
**Glucose (mg/dL)**	(515, 23776958) 112.01 (108.19,115.83)	(521, 24394231) 107.64 (105.14,110.14)	(527, 26145900) 105.12 (102.06,108.18)	(523, 25377294) 103.42 (100.35,106.48)	0.0214
TD					<0.0001
Yes	(149, 23776958) 27.10 (23.41,31.14)	(110, 24394231) 22.69 (18.21,27.91)	(91, 26145900) 15.53 (11.57,20.53)	(79, 25377294) 13.56 (9.53,18.95)	
No	(366, 23776958) 72.90 (68.86,76.59)	(411, 24394231) 77.31 (72.09,81.79)	(436, 26145900) 84.47 (79.47,88.43)	(444, 25377294) 86.44 (81.05,90.47)	
Hypertension					<0.0001
Yes	(236, 23776958) 41.70 (36.50,47.09)	(204, 24394231) 38.04 (32.38,44.05)	(171, 26145900) 29.81 (23.67,36.79)	(125, 25377294) 23.04 (17.41,29.84)	
No	(279, 23776958) 58.30 (52.91,63.50)	(317, 24394231) 61.96 (55.95,67.62)	(356, 26145900) 70.19 (63.21,76.33)	(396, 25377294) 76.84 (70.02,82.49)	
Don’t know	(0, 23776958) 0.00 (0.00,0.00)	(0, 24394231) 0.00 (0.00,0.00)	(0, 26145900) 0.00 (0.00,0.00)	(2, 25377294) 0.12 (0.03,0.50)	
Diabetes					<0.0001
Yes	(102, 23776958) 16.94 (13.59,20.90)	(77, 24394231) 10.60 (7.92,14.05)	(41, 26145900) 6.51 (4.49,9.36)	(25, 25377294) 3.40 (1.79,6.38)	
No	(405, 23776958) 80.64 (76.26,84.37)	(430, 24394231) 87.30 (83.21,90.50)	(466, 26145900) 90.39 (86.83,93.07)	(485, 25377294) 94.27 (90.37,96.65)	
Borderline	(8, 23776958) 2.43 (1.10,5.27)	(14, 24394231) 2.10 (1.02,4.26)	(20, 26145900) 3.09 (1.69,5.61)	(13, 25377294) 2.33 (0.99,5.40)	

Data in the table: For continuous variables: (N-observe, N-represent) survey-weighted mean (95% CI); For categorical variables: (N-observe, N-represent) survey-weighted percentage (95% CI).

MIMS, monitor-independent movement summary; RIP, ratio of family income to poverty; BMI, body mass index; HDL, high-density lipoprotein; TD, testosterone deficiency.

### The association between physical activity and testosterone deficiency

**Table 2** shows the ORs and 95% CIs of the association between physical activity and TD in the three regression models. In both Model I and Model II, daily MIMS units showed negative correlations with TD (all P <0.05). However, in the fully adjusted Model III we found that daily MIMS units did not correlate with TD (OR = 0.97, 95% CI: 0.93, 1.01; P = 0.0889). We further examined the differences in the risk of TD between the different daily MIMS units quartile groups. It was found that in Model III, the ORs of the daily MIMS units group Q3 (0.59, 95% CI: 0.41, 0.86) was significantly different compared to the group Q1 (P <0.05), but the Q4 group did not show such variability (OR = 0.64, 95% CI: 0.37, 1.09; P = 0.0923).

**Table 2:** Weighted multivariate regression analysis of daily MIMS units (×10^3^) with testosterone deficiency.

Exposure	Model I OR(95%CI) P-value	Model II OR(95%CI) P-value	Model III OR(95%CI) P-value
Daily MIMS units	0.94 (0.90, 0.97) 0.0012	0.95 (0.92, 0.98) 0.0064	0.97 (0.93, 1.01) 0.0889
Daily MIMS units quartiles			
Q1 (<9.29)	1.0	1.0	1.0
Q2 (9.29≤ ~ <11.88)	0.79 (0.58, 1.07) 0.1434	0.79 (0.58, 1.06) 0.1269	0.86 (0.59, 1.24) 0.3907
Q3 (11.88≤ ~ <14.78)	0.49 (0.34, 0.71) 0.0007	0.54 (0.38, 0.76) 0.0019	0.59 (0.41, 0.86) 0.0084
Q4 (≥14.78)	0.42 (0.27, 0.66) 0.0007	0.49 (0.32, 0.77) 0.0048	0.64 (0.37, 1.09) 0.0923
P for trend	0.73 (0.64, 0.84) 0.0001	0.78 (0.68, 0.89) 0.0009	0.84 (0.73, 0.96) 0.0216

Model I adjust for: none.

Model II adjust for: age, race.

Model III adjust for: age, race, BMI, HDL, glucose, hypertension, diabetes.

**Figure 2:**
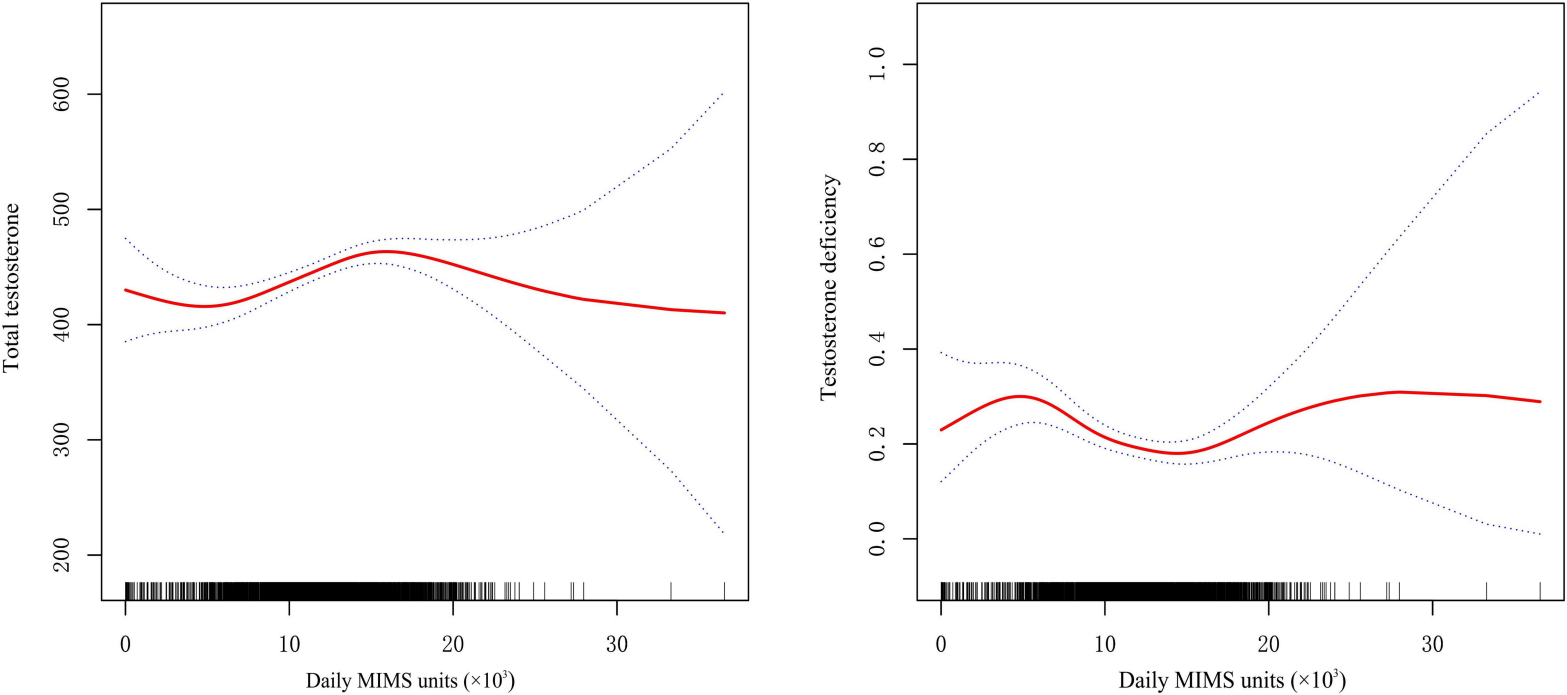
General additive models demonstrate the U-shaped relationship between daily MIMS units and total testosterone and testosterone deficiency (adjust for age, race, BMI, HDL, glucose, hypertension, diabetes).

A U-shaped relationship between daily MIMS units (×10^3^) and risk of TD was observed by spline smoothing fitting with an adjustment for age, race, BMI, HDL, glucose, hypertension and diabetes (**Figure 2**). A turning point value of daily MIMS units [14.77(×10^3^)] was found by segmentation regression model between daily MIMS units and risk of TD. The odds of risk for TD decreased by 0.05 folds in patients per unit increase of daily MIMS units when daily MIMS units <14.77 (×10^3^) (adjusted OR = 0.95, 95%CI: 0.91, 0.99), but increased by 0.12 folds per unit increase of daily MIMS units when daily MIMS units ≥14.77 (×10^3^) (adjusted OR = 1.12, 95%CI: 1.01, 1.23). (LRT test: P <0.01, it demonstrated a non-linear relationship between daily MIMS units and risk of TD) (**Table 3**).

**Table 3:** Weighted threshold effect analysis of daily MIMS units on testosterone using segmented regression.

Daily MIMS units (×10^3^)	Crude OR(95%CI) P-value	Adjusted OR(95%CI) P-value
Total testosterone		
<14.77	5.68 (2.36, 9.00) 0.0022	3.83 (0.27, 7.38) 0.0357
≥14.77	-4.14 (-12.07, 3.80) 0.3153	-6.16 (-14.18, 1.87) 0.1206
Testosterone deficiency		
<14.77	0.94 (0.90, 0.97) 0.0019	0.95 (0.91, 0.99) 0.0205
≥14.77	1.08 (0.98, 1.18) 0.1270	1.12 (1.01, 1.23) 0.0287

Crude: no adjustment.

Adjusted: adjust for age, race, BMI, HDL, glucose, hypertension, diabetes.

In sensitivity analyses, the threshold effect of daily MIMS units on risk of TD was also similar according to baseline characteristics (P-interaction >0.05) (**Table 4**).

**Table 4:** Weighted association between daily MIMS units and testosterone deficiency according to baseline characteristics.

Daily MIMS units (×10^3^)	<14.77	≥14.77
Age (years) group		
18-36	0.99 (0.92, 1.06) 0.7437	1.10 (0.94, 1.29) 0.2673
37-57	0.97 (0.91, 1.04) 0.4581	0.99 (0.88, 1.12) 0.9192
58-80	0.87 (0.80, 0.94) 0.0024	0.78 (0.52, 1.16) 0.2392
**P-interaction**	0.1003	
BMI status		
Normal or low weight	0.92 (0.84, 1.01) 0.0920	0.96 (0.69, 1.34) 0.8277
Overweight	1.03 (0.95, 1.11) 0.4637	0.95 (0.85, 1.06) 0.3327
Obesity	0.91 (0.85, 0.97) 0.0094	1.09 (0.92, 1.30) 0.3224
Missing data	0.74 (0.35, 1.56) 0.4468	NA
**P-interaction**	0.1898	
Diabetes		
Yes	0.91 (0.81, 1.02) 0.1193	1.11 (0.86, 1.43) 0.4510
No	0.95 (0.90, 1.00) 0.0913	1.00 (0.88, 1.15) 0.9469
Borderline	1.13 (0.87, 1.47) 0.3801	1.14 (0.77, 1.68) 0.5174
**P-interaction**	0.6535	

Each stratification adjusted for the factors (age, race, BMI, HDL, glucose, hypertension, diabetes) except the interaction factor itself.

## Discussion

In this cross-sectional study, the participants ultimately included in the study represented 99694383 adult males in the population as a result of the weighted adjusted analysis. And we explored a U-shaped relationship between physical activity in MIMS units and risk of TD among a nationally representative sample of US adult males. We further revealed a threshold effect based on the testosterone deficiency. The study find that physical activity is negatively related with the risk of TD when daily MIMS units <14.77 (×10^3^) and positively related when daily MIMS units ≥14.77 (×10^3^). And this threshold effect was also similar in all of the subgroup populations. To our knowledge, this was the first epidemiologic study to provide clear evidence of a nonlinear association between physical activity summarized using MIMS units and testosterone deficiency.

Studies have been conducted to investigate the association between various physical activity measures and testosterone. Part of the previous study relied on self-reporting of physical activity intensity to address this relationship. Muller et al. (20) conducted a cross-sectional study that included 400 participants and found that higher physical activity is associated with higher TD levels in men aged 40-80 years. Nevertheless, the physical activity of participants in their study was already assessed before the investigation, which may cause bias (20). Similarly, Steeves et al. (11) performed a study that included 738 participants and explored the relationship between testosterone levels and self-reported physical activity, found that higher physical activity may be associated with a lower incidence of low or low normal testosterone in non-obese males. Although self-reporting is the most cost-effective and simplest method of measuring physical activity, the results of the studies are not sufficiently comparable. Given this situation, other studies have utilized accelerometry measures to obtain the intensity of physical activity. A clinical trial from Japan that included 88 subjects found that a 12-week aerobic exercise intervention increase serum total testosterone, free testosterone, and bioavailable testosterone levels in overweight/obese men. In this study, daily steps and physical activity monitoring during the aerobic exercise sessions were conducted using a triaxial acceleromete (21). Giudice et al. assessed the relationship between daily steps count and serum testosterone with the use of a waist-worn uniaxial accelerometer, and found that patients with the lowest daily step count have a higher chance of hypogonadism (13). Another observational study from the UK that included 28,000 participants revealed that men with high physical activity levels had 3% higher concentrations of total testosterone than men with the lowest after adjustment for BMI (22). But it is also challenging to compare the results of studies using different accelerometers.

Different mechanisms are thought to contribute to the effects of physical activity on testosterone level. Some investigators have assumed that this may be related to increased caloric consumption (23, 24). The hypothalamic-pituitary-gonadal (HPG) axis is essential for maintaining normal testosterone levels. Corona et al. (24) suggested that increasing caloric expenditure (physical activity) are efficacious in restoring an altered HPG axis, as often observed in several metabolic conditions, including obesity and metabolic syndrome. In a rabbit model with metabolic syndrome-associated hypogonadotropic hypogonadism, physical exercise (12 weeks of running) exerts beneficial effects on the HPG axis and on the penis, and leads to the synthesis of testosterone (25). In addition, inflammation, estrogen signaling and glucose related genes that are highly expressed in the metabolic syndrome were significantly reduced in rabbits after exercise training (25). This mechanism has also been confirmed in the rat model where diabetic rats are found to have significantly increased levels of luteinizing hormone (LH) after exercise, leading to testosterone production (26). In addition, previous research on human professional sports activity has shown that physical activity damages muscle fibers, which begins a series of processes that lead to the rebuilding of muscle fibers, which includes the stimulation of an increase in the concentration of anabolic hormones such as testosterone (27). Furthermore, physical activity has been found to significantly alter the cortisol/fat ratio, which affects hepatic secretion of GH and IGF-I, and increases luteinizing hormone (LH) secretion and eventual testosterone production by blocking E2-mediated negative feedback to the hypothalamic-pituitary-adrenal (HPA) axis (28).

In the present study, we further found a threshold effect of physical activity on testosterone deficiency, with high-intensity activity being associated with a risk of testosterone deficiency when the optimal value was exceeded. In fact, alongside the beneficial effects of physical activity, prolonged exercise (also known as over-training exercise) can have a negative impact on testicular function (29). Previous studies have suggested that high-intensity endurance exercise, as well as aerobic and strength exercise, can cause lower testosterone concentrations in males, a situation also characterized as exercise-hypogonadal male condition (EHMC) (30). This situation has also been demonstrated in animal studies. By comparing different intensities of swimming exercise on reproductive dysfunction in mature male albino Wistar rats, Manna et al. found that prolonged high intensity training is negatively correlated with reproductive hormones and semen parameters (31). Similar results were found in another study in a rat model (32). In addition, Wang et al. also identified that Long-term intense physical exercise leads to reduced transcript levels of gonadotropin-releasing hormone (GnRH) and kisspeptin (Kiss) in obese mice, finally suppressing testosterone production (33). Current research suggests that the mechanism of inhibition of gonadal function by intense activity may originate centrally, a hypothesis that has been demonstrated in females (34, 35). Low energy availability is thought to potentially mediate this mechanism, and this damaging effect is also collectively referred to as relative energy deficiency in sport (RED-S) (29). Wong et al. found that energy-deprived young healthy lean men can exhibit hypogonadotropic hypogonadism and lower total testosterone, and that serum testosterone levels increased significantly when weight was restored by energy supplementation (36). And this negative effect may be even more pronounced among military personnel, who, due to their occupational peculiarities, may be chronically under-energized, especially during combat training and field exercises, ultimately displaying RED-S (37). Friedl et al. conducted a cohort study analysis and found that volunteers in the energy intake restricted group experienced a significant decrease in testosterone levels to near castration levels after participating in a short-term military training (8-week U.S. Army Ranger training course), which was able to be rapidly restored by energy supplementation, whereas the higher energy intake group experienced a nonsignificant decrease in testosterone after the same training (38). In addition, it has also been proposed that the negative effects of low energy availability on androgens are inconsistent between male and female athletes, the main reason for this being the effect of the presence of menstruation, and similarly, the performance of recovery from low energy availability is different for men and women (29). Nevertheless, the actual mechanism behind the ability of high-intensity physical activity to suppress gonadal function is still largely unknown (39).

Our study has some limitations. This cross-sectional study was based on a nationally representative sample of US adult males in which physical activity and testosterone measurements were collected at the same time point, therefore causality cannot be obtained. In addition, due to the limitations of the NHANES database, we can only define testosterone deficiency as total testosterone below 300 ng/dL. However, in the clinical setting testosterone deficiency is not a simple biochemical indicator and other clinical conditions should be taken into account. Lastly, the high number of missing values in the participant’s smoking data led us to remove this data from the baseline characteristics.

Regardless of these limitations, our study has several strengths. Because different studies may use different accelerometer devices and device-specific algorithms to process accelerometer data, it is challenging to compare the results of these studies. In our study, we used MIMS units to assess activity intensity, a novel physical activity metrics developed using raw data collected by accelerometers through non-proprietary open-source and device-independent algorithms. Therefore, the MIMS unit may contribute to the harmonization efforts across different studies. We further performed a series of sensitivity analyses, and found that the threshold effect of physical activity on testosterone deficiency was also similar in all of the subgroup populations. In addition, the sample size in this study was adequate, which makes our conclusions more reliable and can provide new insights into the management of male reproductive health.

## Conclusions

Using data from a nationally representative sample of US adult males, we found that light to moderate intensity physical activity is associated with a lower odds of testosterone deficiency, while high-intensity physical activity is associated with a higher odds of testosterone deficiency, as characterized by the use of the novel MIMS unit metrics. Furthermore, in our data, this association remains stable across subpopulations, but which needs to be further verified in future investigations using longitudinal data.

## Data availability statement

The raw data supporting the conclusions of this article will be made available by the authors, without undue reservation.

## Ethics statement

The studies involving humans were approved by The Research Ethics Review Board of the National Center for Health Statistics (NCHS). The studies were conducted in accordance with the local legislation and institutional requirements. The participants provided their written informed consent to participate in this study.

## Author contributions

WX: Conceptualization, Writing – original draft. WC: Data curation, Formal analysis, Writing – original draft. YC: Data curation, Formal analysis, Software, Writing – original draft. SW: Conceptualization, Investigation, Methodology, Writing – original draft.
